# Development of Vasoinhibin-Specific Monoclonal Antibodies

**DOI:** 10.3389/fendo.2021.645085

**Published:** 2021-04-20

**Authors:** Nils Müller, Juan Pablo Robles, Magdalena Zamora, Johannes Ebnet, Hülya Markl-Hahn, Gonzalo Martínez de la Escalera, Carmen Clapp, Thomas Bertsch, Jakob Triebel

**Affiliations:** ^1^ Institute for Clinical Chemistry, Laboratory Medicine and Transfusion Medicine, Nuremberg General Hospital & Paracelsus Medical University, Nuremberg, Germany; ^2^ Instituto de Neurobiología, Universidad Nacional Autónoma de México (UNAM), Querétaro, Mexico

**Keywords:** vasoinhibin, prolactin (PRL), monoclonal antibodies, 16K PRL, ELISA - enzyme-linked immunosorbent assay

## Abstract

Vasoinhibin is a protein hormone with antiangiogenic, antivasodilatatory, and antivasopermeability effects generated by the proteolytic cleavage of prolactin. The discovery of its role in diabetic retinopathy and peripartum cardiomyopathy led to the evaluation of new pharmacological treatments in clinical interventional trials. However, the quantitative evaluation of vasoinhibin in biological samples from patients has not been possible due to the lack of vasoinhibin-specific antibodies. Recently, loop 1 of vasoinhibin was identified to have a different three-dimensional structure compared to PRL, and thus to contain vasoinhibin-specific epitopes. Here, we report the development of two sets of vasoinhibin-specific monoclonal antibodies against two neighboring regions of the vasoinhibin loop 1. An experimental sandwich ELISA with two monoclonal anti-vasoinhibin antibodies was developed, which had no cross-reactivity to recombinant human full-length prolactin. The ELISA had a quantitation limit of 100 ng/ml, and intra-assay- and inter-assay coefficients of variation of 12.5% and 14%, respectively. The evaluation of 15 human serum samples demonstrated concentrations of below limit of detection (n=3), below limit of quantitation (n=1) and between 0.23 µg/ml (230 ng/ml) to 605 µg/ml (n=12) in the quantifiable range. Despite the high specificity of the monoclonal-monoclonal antibody sandwiches which discriminate vasoinhibin from PRL, there might be cross-reactivities by serum proteins other than vasoinhibin. A fully established vasoinhibin ELISA may support diagnostic and therapeutic measures in vascular diseases.

## Background

Vasoinhibin (Vi), historically also known as 16 kDa prolactin or 16K PRL, is a protein hormone with antiangiogenic, antivasodilatatory, and antivasopermeability effects generated by the proteolytic cleavage of prolactin (PRL) (1). The regulation of its generation and activity occurs at the hypothalamic, the pituitary, and the target tissue levels, and, due to its correspondence with the three levels of control which define other endocrine axes, this organizational principle was described as the prolactin/vasoinhibin axis (2). Vasoinhibin is generated by the proteolytic cleavage of PRL by cathepsin D (3), matrix metalloproteinases (4), and other proteases in various tissues, including the pituitary gland (5), the human endothelium (6, 7), the placenta (8), the cartilage (4), the retina (9) and the heart (10). Vasoinhibin signals through a still-unidentified receptor on endothelial cells distinct from the PRL-receptor and has various binding partners to mediate its diverse effects (11–13). The discovery of a dysregulation of vasoinhibin generation in diabetic retinopathy and peripartum cardiomyopathy led to the development of new pharmacological treatments to increase or decrease vasoinhibin generation and their evaluation in clinical interventional trials (ClinicalTrials.gov Identifier: NCT03161652 and NCT00998556) (14–16).

The detection and quantification of vasoinhibin in human serum or plasma samples is technically difficult, and the only established technique is immunoprecipitation in combination with western blotting using anti-PRL antibodies. Disadvantages of this technique include limited reproducibility, high inter-laboratory variability, undetermined sensitivity and the lack of precise quantitative information. Clinical studies addressing a change in circulating or local vasoinhibin levels have used of immunoprecipitation and western blotting for semi-quantitative analyses (10, 17, 18), however, owing to the lack of a quantitative vasoinhibin assay, the precise levels in the circulation or elsewhere are unknown. Among the technical options for a quantitative vasoinhibin assay is an enzyme-linked immunosorbent assay (ELISA), the development of which requires antibodies able to discriminate between vasoinhibin and PRL. Such antibodies are not commercially available and in-house antibodies directed against vasoinhibin still bind PRL (19).

**Figure 1:**
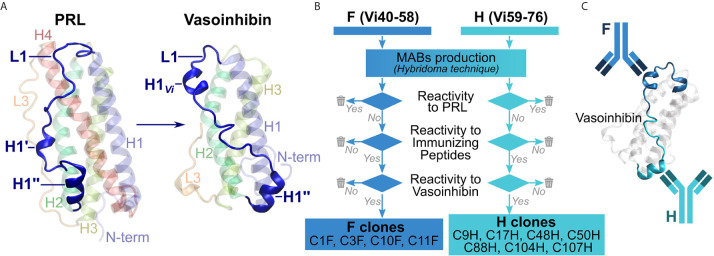
Overview of the antibody development process. **(A)** A suitable, vasoinhibin-specific epitope at the loop 1 region (L1) was identified on the basis of a three-dimensional model of vasoinhibin as reported by Robles et al. **(B)** Antibodies obtained after immunization with two immunizing peptides comprising neighboring L1 regions were selected by their affinity to PRL, to the immunizing peptides, as well as to coated vasoinhibin antigen. **(C)** A sandwich ELISA consisting of two monoclonal, vasoinhibin-specific antibodies was set up.

Unlike for full-length PRL (20, 21), an experimental three-dimensional structure for vasoinhibin is not available, also because aggregation of the vasoinhibin molecule prevented resolution of its structure by NMR (22). Information on potential epitopes unique to vasoinhibin emerged from the recently reported three-dimensional model of vasoinhibin, which revealed significant conformational differences with full-length PRL in the area within the loop 1 (L1) comprising amino acids 40 – 76 (23). This region appeared to be a new epitope created only after the loss of the 4^th^ helix that follows the proteolytic cleavage of PRL and, thereby, a suitable region for the generation of monoclonal anti-vasoinhibin antibodies that do not react with PRL (**Figure 1A**). Here, we report the development of monoclonal vasoinhibin antibodies which bind to two distinct and unique epitopes of the vasoinhibin molecule, and their evaluation in experimental ELISA applications.

## Methods

### Production of Anti-Vasoinhibin Specific Monoclonal Antibodies

The production of the monoclonal antibodies was performed by Davids Biotechnologie, Regensburg, Germany. Briefly, two immunizing peptides, comprising amino acid residues 40-58 of mature PRL, herein abbreviated Vi40-58, [FDKRYTHGRGFITKAINSC, abbreviated FDK, or F in the antibody designation, the use of this peptide for the generation of antibodies is protected by a pending international patent (24)], and amino acid residues 59-76, herein abbreviated Vi59-76, (HTSSLATPEDKEQAQQMN, abbreviated HTS, or H in the antibody designation) were chemically synthesized and conjugated to a keyhole limpet hemocyanin (KLH) carrier. Four BALB/c mice were injected with the 75 µg of the FDK peptide and another four mice with the HTS peptide for immunization. The immunization was repeated several times over 98 days under monitoring of titer levels. The animal with the highest titer in each group was sacrificed, spleen cells were isolated and fused with myeloma cells. Clones producing antibodies reactive against recombinant human PRL in an indirect ELISA (wells coated with PRL) were eliminated. Clones producing antibodies non-reactive to PRL were then tested for affinity to their respective immunizing peptide and if positive, were subcloned employing the limited dilution method. The antibodies were then tested for their affinity with coated vasoinhibin (Vi1-146) and discharged when no affinity was found. Four antibodies against Vi40-58 (C1F, C3F, C10F, C11F) and seven antibodies against Vi59-76 (C9H, C17H, C48H, C50H, C88H, C104H, C107H), which passed the antibody selection procedure (**Figure 1B**) were obtained from the production (the use of the antibodies for the development of diagnostic methods is protected by a pending international patent). The production of the antibody C1F was suspended after the first batch due to the presence of heterogeneous immunoglobulin subclasses. Biotinylation of the antibodies was performed with an antibody biotinylation kit for immunoprecipitation (Thermo Fisher Scientific, Rockford, IL, cat. no. 90407) according to the instructions of the manufacturer. Sixty-four µg of antibody was biotinylated with 2 µl biotin-solution in a total volume of 100 µl PBS, resulting in an end concentration of the biotinylated antibody of 0.64 µg/µl.

### Indirect ELISA of Coated Recombinant Vasoinhibin and PRL

Clear, flat-bottom, 96-well plates (Brand GmbH, Wertheim, Germany, cat. no. 781722) were used. For the indirect ELISA, recombinant vasoinhibin and PRL were diluted to a concentration of 1 µg/ml in 100 µl carbonate buffer (0.55 g sodium carbonate anhydrous, 1.24 g sodium hydrogen carbonate), added to the wells and incubated overnight to allow coating with the antigen. After the coating, the wells were blocked with 250 µl BSA (Blocker BSA in TBS (10X) Concentrate, Thermo Fisher Scientific, cat. no. 37520) for 3 h while shaking. Afterwards, 100 µl of TBST with 1.1 µg/ml of the anti-vasoinhibin antibodies was incubated overnight at 4°C. The secondary HRP-conjugated goat-anti-mouse (heavy and light chain) antibody (Jackson ImmunoResearch Laboratories, Ely, UK, cat. no. 115-035-062) was diluted 1:5000 in 100 µl TBST, added to the wells, and incubated for 1 h at RT while shaking. Between each step, washing of the plates with 300 µl tris-buffered saline with 0.05% Tween (TBST) was performed with an automatic washer (ELx50 Microplate Strip Washer, Biotek, Bad Friedrichshall, Germany). For the detection, 100 µl 3,3′,5,5′-Tetramethylbenzidine (TMB)-solution (1-Step Ultra TMB-ELISA Substrate Solution, Thermo Fisher Scientific, cat. no. 34028) was added to the wells and incubated for 15 min. Without a washing step, 100 µl stop-solution was added and the plate was read at 450 nm (Synergy 2 Multi-Mode Microplate Reader, Biotek). Standard curves were measured in duplicates, and all other samples in triplicates. The results are representative of three or more independent experiments.

### Sandwich ELISA of Recombinant Vasoinhibin and PRL in Solution, and of Vasoinhibin in Human Sera

For the sandwich ELISA (**Figure 1C**), the plates were coated with 100 µl of 1.1 µg/ml antibodies in carbonate buffer, and incubated overnight at 4°C. The blocking of the wells was performed the same way as for the indirect ELISA. Afterwards, various concentrations of vasoinhibin, PRL, human serum (diluted up to 1 to 20 in TBST) and BSA as blank were dissolved in 100 µl of TBST, added to the wells and incubated for 1 h at RT while shaking. The bound antigen was detected by the biotinylated antibodies, which were diluted 1 to 3000 in 100 µl TBST overnight at 4°C. Streptavidin-poly-HRP (Thermo Fisher Scientific, 21140) was added diluted 1 to 3000 in 100 µl TBST for 1 h while shaking. The detection was performed the same way as for the indirect ELISA. The standard curves were fitted on the basis of the 4-parameter Rodbard equation. Cross-titration was performed to determine the most efficient caption and detection antibody concentration. Therefore, a sandwich ELISA with a constant antigen concentration of 5 µg/ml, various caption antibody concentrations (1, 2, 3, 4, 5, 6 µg/ml), and various detection antibody dilutions (1 to 1000; 1 to 2000, 1 to 3000, 1 to 4000) was performed. PRL in human sera was determined by a commercial ELISA kit according to the instructions of the manufacturer (cat. no. EIA-1291, DRG Diagnostics, Marburg, Germany).

### Recombinant Human Vasoinhibin and PRL Preparations

For microplate assays, a recombinant human vasoinhibin (source: *E. coli* expression), comprising the first 147 amino acid residues of the mature wt PRL sequence (UniProt ID: P01236) was used (custom production, Giotto Biotech, Florence, Italy). Recombinant human PRL, expressed in HEK cells, was used (Sigma Aldrich, cat. no. SRP9000).

## Results

### Selection of Anti-Vasoinhibin Monoclonal Antibodies Able to Discriminate Between the Vasoinhibin and PRL Standards in an Indirect ELISA

**Figure 2:**
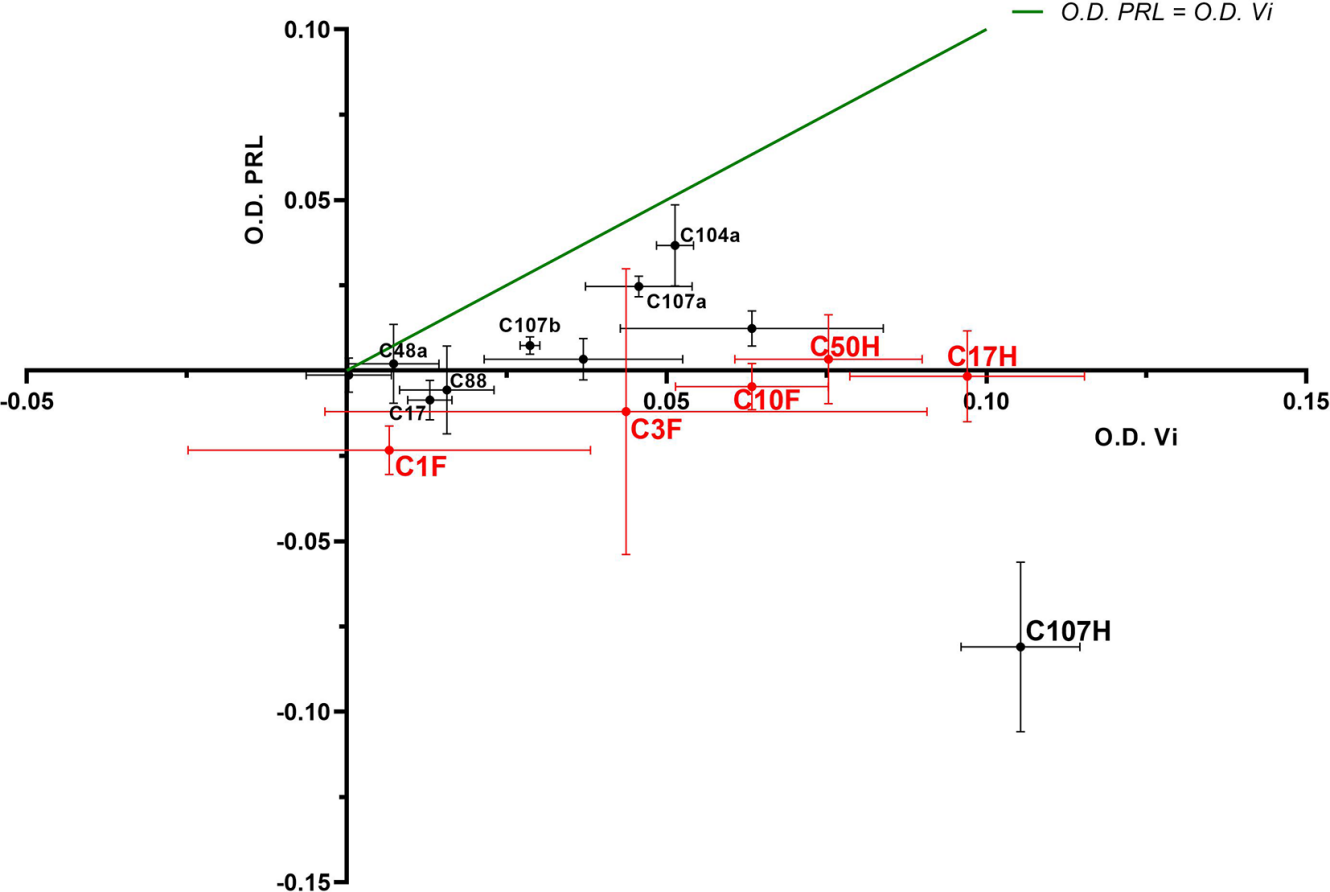
ELISA of coated vasoinhibin and PRL with vasoinhibin-specific monoclonal antibodies. Four FDK-antibodies and seven HTS-antibodies were tested in a semi-quantitative indirect ELISA for their specificity to detect coated vasoinhibin in comparison to coated full-length PRL. The affinity of each antibody with vasoinhibin represented by its O.D. is plotted on the x-axis, and the affinity of each antibody with PRL by its O.D. on the y-axis, each antibody is indicated as a cross with mean and standard deviation, derived from triplicate measurements. The center point of each cross is the mean O.D. value of the triplicate measurement, and the lines show the range of the standard deviation. The green line represents the signal localization of the cross centers in case of equal affinity against vasoinhibin and PRL. The selected antibodies are shown in red. All tested antibodies are right of the green line toward the x-axis, which demonstrates a preference of vasoinhibin over PRL.

Four FDK-clone antibodies (C1F, C3F, C10F, C11F) and seven HTS-clone antibodies (C9H, C17H, C48H, C50H, C88H, C104H, C107H) were tested in a semi-quantitative indirect ELISA for their specificity to detect the vasoinhibin coating the plate in comparison with plates coated with full-length PRL. The antibody affinity (a cross with mean and standard deviation) with vasoinhibin and PRL was plotted on an x/y-axis graph, where the y-axis represents PRL- and the x-axis vasoinhibin-reactivity and a theoretical line was plotted, in which PRL affinity equals vasoinhibin affinity. All tested antibodies demonstrated a preference of vasoinhibin over PRL (**Figure 2**), which was an expected result as clones with reactivity to coated PRL were already deselected in a first round of specificity testing. The affinity of the antibodies C1F, C3F, C10F, C17H, C50H against vasoinhibin showed a relatively strong signal whereas their affinity towards PRL was nearly absent and similar to the wells containing neither protein. These antibodies were selected for their use in a sandwich ELISA.

### Selection of Anti-Vasoinhibin Monoclonal Antibodies Able to Discriminate Between the Vasoinhibin and PRL Standards in a Sandwich ELISA

**Figure 3:**
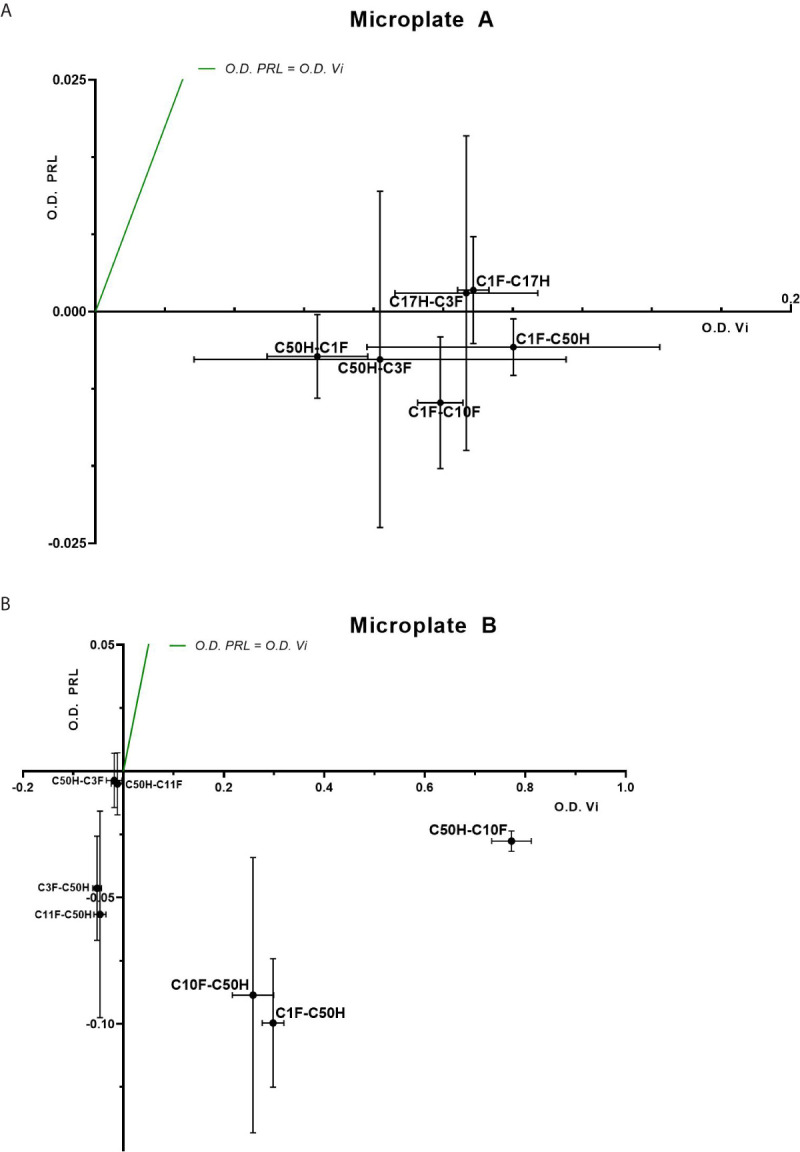
Sandwich ELISA of vasoinhibin and PRL with vasoinhibin-specific monoclonal antibodies. Plots of sandwich ELISA with anti-vasoinhibin monoclonal antibodies showing the affinity with vasoinhibin (1.0 µg/ml) (x-axis) and PRL (1.0 µg/ml) (y-axis). The center point of each cross is the mean O.D. value of the triplicate measurement, and the lines show the range of the standard deviation. The green line represents the signal localization of the cross centers in case of equal affinity against vasoinhibin and PRL. **(A)** shows sets of antibody pairs tested on Microplate A, each pair is indicated as a cross with mean and standard deviation. **(B)** shows the affinity of another set of antibody pairs on Microplate B with suitable affinities that do not recognize PRL. Affinities in the negative scale of the PRL y-axis are a result of wells containing PRL standard being even less reactive than blank wells. Sandwiches C10F-C50H, C1F-C50H, and C50H-C10F show high affinity to vasoinhibin and no affinity to PRL.

**Figure 4:**
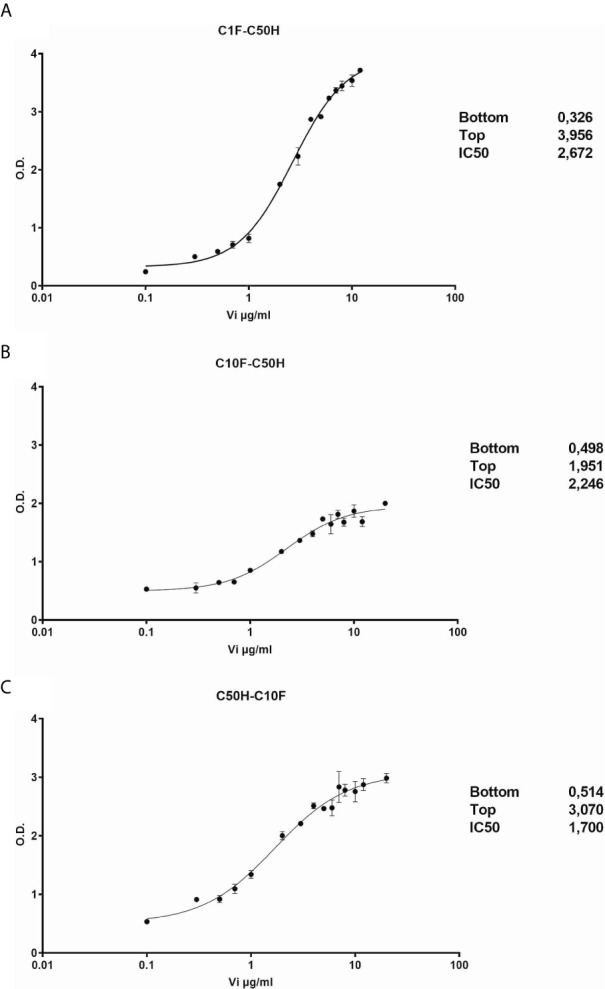
Vasoinhibin ELISA standard curves. Sandwich ELISA standard curves of antibody pairs C1-C50 **(A)**, C50H-C10F **(B)** and C10F-C50H **(C)** against recombinant human vasoinhibin standard with the interpolated Rodbard curve with BLK-subtracted values. The O.D. at the bottom and the top of each curve is indicated. IC50 values are the analyte concentrations at the O.D. halfway between the top and the bottom.

**Figure 5:**
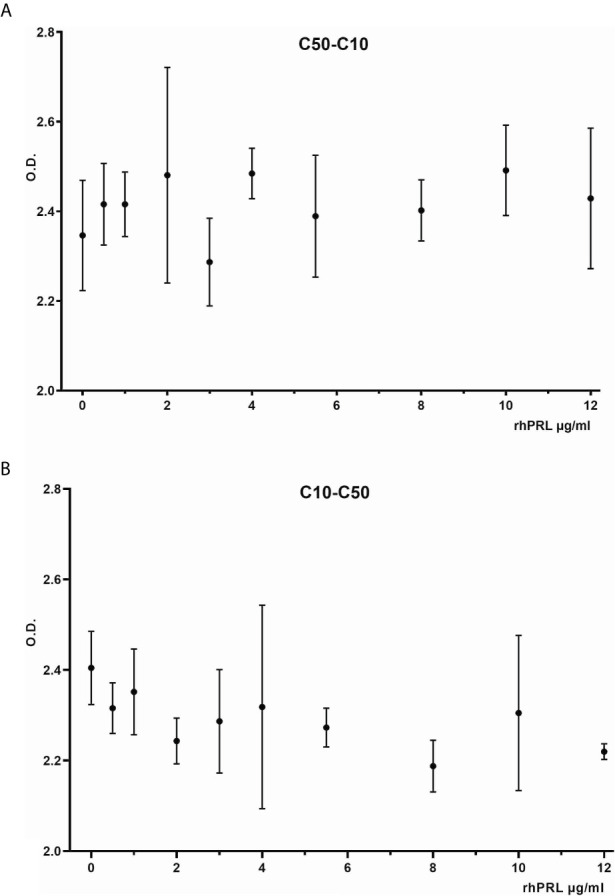
Vasoinhibin ELISA in presence of PRL. Sandwich ELISA of recombinant human vasoinhibin standard in the presence of various concentrations of PRL standard using sandwich C50H-C10F **(A)** and C10F-C50H **(B)**.

The antibodies selected were combined as capture and detection antibodies to unveil combinations with good performance. The results of this screening procedure are shown in **Figures 3A, B**. The useful antibody combinations are those furthest right from the green line (a theoretical line plotted, in which PRL affinity equals vasoinhibin affinity). The pairs C1F-C50H and C50H-C10F/C10F-C50H appeared particularly suited (**Figure 3B**), and the cells producing these clones were used for the production of larger quantities. Repeated testing showed, that C1F and C50H and C10F and C50H were suitable both, as caption and detection antibodies for each antibody pair, respectively. Sandwich ELISA standard curves using recombinant vasoinhibin concentrations ranging from 0.1 to 20 µg/ml were established for the C1F-C50H (**Figure 4A**), C10F-C50H (**Figure 4B**) and the C50H-C10F (**Figure 4C**) antibody pairs. A quantitation limit of 100 ng/ml (lowest calibration standard) was set, and intra-assay- and inter-assay coefficients of variation were 12.5% and 14%, respectively. The measurement of a constant concentration of vasoinhibin (5.5 µg/ml), using antibody pairs C50H-C10F (**Figure 5A**) and C10F-C50H (**Figure 5B**) was not affected in the presence of 8 different concentrations of PRL, as the % deviation of vasoinhibin signals in the presence of PRL compared to vasoinhibin only was smaller than the intra- and inter-assay coefficients of variation (**Table 1**). The ELISA performance characteristics are summarized in **Table 1**.

**Table 1:** ELISA performance characteristics.

Vasoinhibin standard curve in all sandwiches					0.1 – 20 µg/ml	
Applied LOQ (lowest calibration standard)					100 ng	
Intra-assay coefficient of variation of standard					12.5%	
Inter-assay coefficient of variation of standard					14%	
Cross-reactivity with PRL at 12 µg/ml at 5.5 µg/ml vasoinhibin standard (% deviation compared to vasoinhibin standard only)					C50-C10 = 3.3% C10-C50 = 7.3%	
Sandwich	BLK [O.D.] (mean ± SD)	LOD [O.D.]	LOD - BLK [O.D.]	LOQ [O.D.]		LOQ – BLK [O.D.]
C1F-C50H	0.096 ± 0.011	0.129	0.033	0.206		0.110
C10F-C50H	0.768 ± 0.036	0.876	0.108	1.128		0.360
C50H-C10F	0.725 ± 0.071	0.938	0.213	1.435		0.710

BLK, blank; LOD, limit of detection; BLK mean plus the three-fold standard deviation of blank, LOQ, limit of quantitation, the applied limit of quantitation was the lowest calibration standard of 100 ng, the LOQ is the BLK-mean plus the 10-fold standard deviation of blank. LOD and LOQ are also shown as BLK-subtracted values for comparison with the standard curves.

### Evaluation of Serum Samples Using a Sandwich ELISA With Vasoinhibin-Specific Monoclonal Antibodies

The application of the vasoinhibin ELISA to evaluate a collection of 15 human serum samples demonstrated a wide variability of O.D. values representing the affinity between the capture- and detection-antibody, and serum proteins (**Table 2**). When this affinity is converted to concentrations, the O.D. corresponds to below LOD (n=3), below LOQ (n=1) and between 0.23 µg/ml (230 ng/ml) to 605 µg/ml (n=12) in the quantifiable range. PRL levels in these samples were normal, ranging from 4.8 – 23.9 ng/ml (**Table 2**). There was no significant correlation between PRL concentrations and O.D. values of the vasoinhibin ELISA (spearman r = 0.09).

**Table 2:** Blank-subtracted O.D. values and the corresponding vasoinhibin (Vi) concentrations using monoclonal anti-vasoinhibin sandwich ELISA in a variety of human serum samples.

Sample	Serum ID	C10-C50, S1	C10-C50, S2	C1-C50, S1	C1-C50, S2	Vi-concentration	PRL concentration
		O.D., mean ± SD				µg/ml	ng/ml
1	3780-146-8	3.660 ± 0.115	3.711 ± 0.035	3.464 ± 0.155	3.545 ± 0.062	>STD/605.85	15.6
2	2866-146-22	0.073 ± 0.006	0.358 ± 0.008	-0.014 ± 0.001	0.014 ± 0.001	0.35/<LOD	10.1
3	3780-146-9	–	0.502 ± 0.016	0.550 ± 0.032	0.634 ± 0.025	2.54/25.48/26.48	7.8
4	3780-146-1	0.530 ± 0.020	–	0.045 ± 0.006	0.073 ± 0.009	3.24/<LOD	7.4
5	2869-146-5	0.327 ± 0.090	–	0.013 ± 0.001	0.037 ± 0.002	1.75/<LOD	5.1
6	3780-146-2	–	2.868 ± 0.078	0.516 ± 0.036	0.692 ± 0.049	41.63/24.08/29.09	23.9
7	3780-146-21	0.717 ± 0.012	–	0.080 ± 0.010	0.116 ± 0.005	4.55/1.77/2.25	10.0
8	2866-146-25	–	3.197 ± 0.035	0.711 ± 0.017	0.897 ± 0.036	64.61/43.27/38.86	4.4
9	3780-146-5	–	2.048 ± 0.013	0.334 ± 0.020	0.368 ± 0.015	11.46/12.88/17.64	4.8
10	2866-146-23	–	–	0.182 ± 0.003	0.242 ± 0.005	5.49/6.28	7.2
11	3780-146-3	–	–	0.686 ± 0.066	0.839 ± 0.025	36.83/39.0	–
12	2866-146-24	-0.013 ± 0.012	0.039 ± 0.016	-0.014 ± 0.001	0.007 ± 0.001	<LOQ/<LOD	–
13	2866-146-19	–	–	1.858 ± 0.099	2.340 ± 0.045	194.11/227.63	–
14	2869-146-3	0.060 ± 0.010	0.238 ± 0.033	-0.009 ± 0.003	0.014 ± 0.003	0.23/<LOD	–
15	3780-146-20	–	–	-0.008 ± 0.010	0.013 ± 0.001	<LOD	–

Four series (S) of measurements with different dilutions were done, evaluating sandwiches C10-C50 and C1-C50, twice each. O.D. values are not correlated with PRL concentrations (spearman correlation coefficient across all series r = 0.09).

## Discussion

The present paper reports the development of monoclonal antibodies against vasoinhibin which do not bind to full-length human PRL. Such antibodies are not commercially available, and, to our knowledge, have not been developed elsewhere. Here, microplate assays demonstrate specificity of the antibodies for vasoinhibin in the presence of PRL. An important precondition for the development of these antibodies was the recognition of the unique three-dimensional structure in the L1 region of vasoinhibin which does not exist in full-length PRL (23). The vasoinhibin antibodies are suitable for sandwich ELISA applications, and are therefore promising in terms of the development of an ELISA which could be used to quantitatively determine circulating vasoinhibin levels in patients. The fact that circulating vasoinhibin levels are unknown and cannot be monitored is a significant barrier for the translation of the role of the prolactin/vasoinhibin axis in health and disease. It is also a safety concern in interventional trials in which the generation of vasoinhibin is pharmacologically interfered with, for example in the trials evaluating blocking vasoinhibin generation in patients with peripartum cardiomyopathy or stimulating it in patients with diabetic retinopathy (15).

### Evaluation of Serum Samples Using a Sandwich ELISA With Vasoinhibin-Specific Monoclonal Antibodies

The range of vasoinhibin affinity shown here, with O.D. values of 15 samples corresponding to concentrations of below LOD, below LOQ and between 0.23 µg/ml (230 ng/ml) to 605 µg/ml, is consistent with reported vasoinhibin levels determined by immunoprecipitation and western blotting in terms of high inter-individual variance, non-detectable levels and levels equal to or above PRL levels (25). The high O.D. measured in some of the samples that indicates levels in the µg-range, however, appears too high given the low-ng range for PRL, and is therefore awaiting evaluation during further serum ELISA development. For example, PRL-immunoreactive proteins in the serum, which appear not to be 23 kDa PRL, such as the high abundant 28 kDa protein in human sera, which may indicate a vasoinhibin dimer (26, 27), should be identified and tested for their affinity with the vasoinhibin-specific monoclonal antibodies. Also, the issue of immunoglobulin cross-reactivity with anti-PRL antisera (28) requires attention, and an interference between the vasoinhibin-specific monoclonal antibodies and immunoglobulins should be excluded.

## Conclusion

The present report communicates the development of monoclonal antibodies against human vasoinhibin which show no cross-affinity to full-length PRL. These antibodies appear suitable for the development of an ELISA to determine vasoinhibin levels in serum and other body fluids. Despite the high specificity of the monoclonal-monoclonal antibody sandwiches which discriminate vasoinhibin from PRL, it is not yet clear whether there might be cross-reactivities by serum proteins other than vasoinhibin. Such uncertainties should be addressed with the full set of experiments required for the development of a serum ELISA for clinical purposes, including establishing a standard curve in vasoinhibin-depleted serum at low concentration ranges, immunoprecipitation experiments of human serum with capture- and detection-antibodies including semi-quantitative evaluation of precipitates and comparison with ELISA measurements, the evaluation of vasoinhibin-spiked serum samples, a higher sensitivity of the assay, a better characterization of endogenous vasoinhibin isoforms, as well as evaluation of vasoinhibin levels in a larger number of serum samples of patients from well-defined clinical cohorts.

## Author’s Note

A poster was presented at the 22^nd^ European Congress of Endocrinology, e-ECE 2020, September 5 – 9, 2020, DOI: 10.1530/endoabs.70.AEP543.

## Data Availability Statement

The raw data supporting the conclusions of this article will be made available by the authors, without undue reservation.

## Ethics Statement

Human studies: human serum samples for research purposes were purchased from BBI Solutions, Cardiff, UK, and were exempt from Title 45, Title 21 and HIPAA/IRB/consent requirements (Receiver and/or the study investigator cannot link private information to the individual from whom the material was obtained). Animal studies: The animal studies have been registered, reviewed and approved by the government of Lower Franconia in Würzburg, Germany.

## Author Contributions

Conception and design of research: JT, NM, JPR, MZ, CC, TB. Performed experiments: NM, JT, JE, HM-H. Analyzed data: NM, JT, MZ, JPR, JE, TB. Interpreted results of experiments: NM, JT, JE, MZ, JPR, GME, CC, TB. Prepared figures: NM, JT, JPR. Wrote manuscript: JT, NM, JPR, MZ, CC. Edited and revised manuscript: GME, JE, TB. All authors contributed to the article and approved the submitted version.

## Funding

Supported in part by grant A1-S-9620B from the National Council of Science and Technology of Mexico (CONACYT) to CC.

## Conflict of Interest

The authors declare the following competing interests: CC, JPR, MZ, GME, JT, and TB are inventors of a pending Mexican (MX/E/2019/079075) and International (PCT/EP2020/069154) patent application covering the use of the anti-vasoinhibin monoclonal antibodies for diagnostic purposes. The Universidad Nacional Autónoma de México (UNAM), and the authors JT and TB are owners of the pending-patent applications.

The remaining authors declare that the research was conducted in the absence of any commercial or financial relationships that could be construed as a potential conflict of interest.
